# A Gender-Based Point of View in Pediatric Neurology

**DOI:** 10.3390/jpm13030483

**Published:** 2023-03-08

**Authors:** Pierluigi Diana, Susanna Esposito

**Affiliations:** Pediatric Clinic, Department of Medicine and Surgery, University Hospital of Parma, 43126 Parma, Italy

**Keywords:** gender, gender dysphoria, gender identity, neurology, neuropsychiatric, pediatrics

## Abstract

While the significance of gender has only recently been recognized, gender assigned at birth has long been understood to have a significant influence on a number of illnesses. Due to the paucity of data in this regard in pediatrics, the purpose of this narrative review is to frame the most recent knowledge about the role of gender assigned at birth in the neurological development and neuropsychiatric disorders among young people. Literature analysis showed that gender disparities exist in neurologic and neuropsychiatric disorders among the pediatric population and supported the fact that new guidelines should take this into account. However, there is an urgent need for specific studies focused on gender role among children and adolescents in order to better understand how this can relate to diagnosis, development and treatment of different neurologic and neuropsychiatric diseases. Moreover, further efforts should be directed to identify unique risks linked to gender disorders and gender dysphoria as well as taking into account a gender point of view when approaching a pediatric patient.

## 1. Background

Physical aspects of gender, such as the appearance of external genitalia or secondary gender characteristics, are referred to as “gender assigned at birth”. Gender includes social gender in the sense of gender presentation, expression and role as well as psychological gender in the sense of gender identity [1,2]. Data on the formation of gender identity are still lacking, but prior research has shown that it appears to be a complicated phenomenon in which epigenetic, biochemical and prenatal variables (including genes and hormones) may all play a role [3,4,5]. According to a very recent review, gender identity and sexual orientation are likely programmed in the brain during early development: during the intrauterine phase in the second half of pregnancy, a surge in testosterone masculinizes the developing male brain; in the absence of this surge, a female brain will develop [6]. While the significance of gender has only recently been recognized, gender assigned at birth has long been understood to have a significant influence on a number of illnesses. Nowadays, medicine is moving towards an individualized approach to the patient that must include both biological sex and gender. Even if the examples of such applications in pediatrics are fewer, it has been shown in other medicinal areas how gender can actually affect the human pathology. As reported by Biskup et al., several studies conducted among adult patients have reported a strong association between gender and neuropsychiatric disorders (such as Alzheimer disease, schizophrenia and depression) [7]. The purpose of this review is to frame the most recent knowledge about the role of gender assigned at birth on the neurological development and neuropsychiatric disorders among young people.

## 2. Methods

This is a narrative review of literature focusing on neurological and neuropsychiatric gender medicine in pediatrics. This review was carried out by the Pediatric Clinic in the University of Parma, Parma, Italy. Systematic searches were performed using PubMed up to January 2023. Language was restricted to English. Search terms included gender, transgender, gender nonconforming, health, neuropsychiatric and neurodevelopment in combination with adolescents, children and/or youth. Original research studies, review articles, letters to the editor and cohort studies published between 2012 and 2023 were included. Data from earlier studies were taken into account if relevant to the scope of this review. All relevant articles were then evaluated, and pertinent articles were included in this review. In this review, we will refer to the terms male, female, boys and girls always in the sense of assigned gender at birth, unless specified differently.

## 3. Brain Development

The human brain is a complex structure that goes through deep remodeling during childhood and adolescence, especially under the hormonal pressure that marks this critical part of youth [8,9,10,11]. This is when several neuropsychiatric symptoms emerge, suggesting that brain development disorders may lead to specific neuropsychiatric conditions. Furthermore, it has been shown that there is a difference in their prevalence between adolescents being assigned as male at birth (AMAB) and assigned as female at birth (AFAB) [12,13,14,15]. Remarkable differences in the central nervous system development among AMAB and AFAB people have been reported. Young and adult AMAB people are shown to have brain sizes about 9–12% larger compared to AFAB people [16,17,18,19,20,21], with both total gray matter volume and gray matter mass greater in an AMAB than in an AFAB [18]. It has also been reported that cortical gray matter positively grows in the pre-adolescent period, then decreases faster in males than in females later in life [17,22,23]; conversely, white matter volume shows a linear increase from childhood to young adulthood [17,22,24]. While an AMAB shows larger white and gray matter, an AFAB shows a greater ratio of gray to white matter [20,25,26,27] and a greater gray matter density across the cortex [20,28]. This has not been confirmed in females younger than 8 years old in other studies [18]. Moreover, a 2011 study performed among children from 6 to 10 years old has reported that older assigned females at birth have greater cortical thickness then their male peers in the right insula and sensory area [29]. Disparities based on gender assigned at birth on white matter development are likely due to different hormonal impacts as seen by Herting et al.; higher testosterone exposition in AMAB youths predicts greater fractional anisotropy in their white matter tracts compared to AFAB youths [30].

Some differences based on gender have been also reported in structural variability. Two studies underlined how assigned males at birth show a greater variance in the hippocampus, pallidum and putamen compared to assigned females at birth [21,31].

As previously reported, there are even disparities based on biological gender of cerebral blood flow in both adults and youths [32,33,34,35,36]. It has been shown that an AMAB youth has lower blood flow in the posterior cingulate and experiences a linear decline in cerebral blood flow from age 8 to 22 years, as opposed to an AFAB [37,38]. Moreover, it has been reported how cerebral blood flow levels seem to be equivalent among early pubescent boys and girls, but then diverge around mid-puberty [38].

## 4. Febrile Seizures

Febrile seizures (FSs) are the most common neurological disorder during childhood. FSs occur in association with a central temperature of 38 °C or higher and in the absence of a history of prior afebrile seizures and without evidence of central nervous system infection [39]. Several risk factors are known to be associated with FSs, such as age <1 year old, a family history of epilepsy or FS, presence of seizures with low fever and AMAB [40]. In a recent study, Kazemi et al. reported a male predominance of FSs, as Mahyar et al. found previously by observing a prevalence of 66% among males [41]. In accordance with those findings, Renda et al. showed a male/female ratio varying between 1.2:1 and 1.7:1. Moreover, a difference in the age of onset between an AMAB and AFAB has been observed, with the most frequent age being around 6–12 months in females and 36 months in males [42]. Table 1 summarizes the available evidence on the relationship between FSs and gender.

## 5. Depression and Anxiety

Anxiety is one of the most common psychiatric disorders, with an estimated lifetime prevalence near to 25% [12,43,44]. It usually breaks up in childhood, increasing in the adolescence period. It is well accepted that an anxiety diagnosis is two times more frequent in an AFAB than in an AMAB, especially during pubertal development [45]. While it is not well documented whether anxiety is associated with brain structural abnormalities or not [46,47], a more consistent relationship with cerebral blood flow has been shown. Several studies have indeed reported a positive association between anxiety symptoms and greater regional cerebral blood flow in the insula and amygdala prefrontal/temporal cortices [48,49,50,51], which may explain why females frequently present more symptoms during puberty when they show an increased cerebral blow flood [52]. Moreover, a study conducted on adults has showed how males and females react differently to a given stressful condition: on the one hand, cerebral blood flow increases in the right prefrontal cortex in an AMAB, with a reduced flow in the left orbital cortex; on the other hand, an AFAB showed an increased flow in the ventral striatum, putamen, insula and cingulate cortex [53].

Furthermore, an AFAB experiences more severe depressive symptoms than an AMAB, starting from adolescence and with a prevalence of major depressive episodes being two times higher in an AFAB than in an AMAB [45]. Moreover, the female biological gender appears to be associated with internalizing symptoms and a higher rate of suicide attempts, while the opposite sex with greater aggressive symptoms, drug abuse, risk-taking behaviors and completed suicide [54,55]. However, it is still not clear how depressive manifestations differ between AMAB and AFAB people. Wise et al. reported smaller prefrontal cortex volumes in females with major depressive disorder [56]. Whittle et al. underlined an association between depression and greater amygdala growth in females; furthermore, they focused on the link between depressive symptoms and smaller nucleus accumbens volumes in girls [57].

Table 2 summarizes the available evidence on the relationship between anxiety/depression and gender.

## 6. Autism Spectrum Disorders and Attention Deficit/Hyperactivity Disorders

Autism spectrum disorder (ASD) is associated with impairments in social and communication skills, repetitive interests and peculiarities in sensitive perception; however, it is well established that they must be considered as a heterogeneous group of conditions [58]. Studies suggest a major role is genetic factors in ASD etiology [59] and show that up to 1% of the population is affected by ASD, mainly prevalent in assigned males at birth [60].

AMAB predominance is a well-established fact in ASD, with a male–female ratio reported in many studies in the range of 3–4:1 [60,61]. Nevertheless, in recent research, the prevalence of ASD in AFAB people compared with AMAB people in several known ASD-related neurological phenotypes has been examined [62]. A significant female representation has been related to microcephaly, developmental regression, neurological dysfunction and seizure frequency, suggesting that abnormal neurological phenotypes are consistently associated with female gender. Some of these findings are in accordance with previous studies. Miles et al. reported a higher rate of microcephaly among AFAB people with ASD [63], and a greater frequency of seizures in AFAB people with ASD has been observed by Bolton et al. [64]. According to Levy et al., AFAB people might be more protected than AMAB people from idiopathic ASD, showing on the other hand a higher prevalence of ASD as a secondary manifestation of other disorders [65].

It is widely reported that also attention-deficit/hyperactivity disorder (ADHD) is much more represented among young AMAB people than in AFAB people (2.4 times greater) and also into adolescence [66]. On the other hand, since the higher rate of misdiagnosis is in girls, it has been described a “gender paradox” which results in more impairment and worse outcomes in AFAB people with ADHD than in AMAB people [67]. Concerning the reasons behind the ADHD sexual disparity, we still have a lack of data in this regard. A reduced gray matter volume in the left lateral promotor cortex has been described in young females with ADHD, in opposition to reduced white matter volume in the prefrontal cortex in AMAB people [68]. Moreover, Dirlikov et al. reported an association between reduced surface area of the prefrontal cortex and the female biological gender, while in young AMAB people, the reduction was restricted to the right cingulate and left medial prefrontal cortex [69]. These data are in contrast with other studies where no differences between male and female youths diagnosed with ADHD have been found [70,71].

Table 3 summarizes the available evidence on the relationship between ASD/ADHD and gender.

## 7. Multiple Sclerosis

Multiple sclerosis (MS) affects the central nervous system and consists in a demyelinating condition due to autoimmune inflammation. Even if its prevalence among adults is higher, it is estimated that a rate between 2.7 and 5.4% of patients develop the disease before 18 years old [72]. In a recent study, two peaks of disease onset have been reported in youths: one at around 4 years of age and another one later at around 15 years [72].

It is well known that MS risk is related to gender assigned at birth: AFAB adults present a higher prevalence of MS, earlier disease onset and slower disability progression [73,74]. Similar data have been recently shown among young patients; after a similar incidence between males and females until the age of 9 years, a strong female predominance appears afterwards, with a peak in the age range 12–17 years, which may suggest a role of sex hormones on the onset of the condition during adolescence [72]. The importance of the hormonal modulation on the age of MS onset has been supported by Lulu et al. by observing how the MS onset in young females peaked 2 years after menarche, with a slightly higher age at onset in girls compared to boys [72,75].

Available evidence on the relationship between multiple sclerosis (MS) and gender is reported in Table 4.

## 8. Primary Eating Disorders

The last ten years have shown increasing attention on the role of gender regarding the development of primary eating disorders (EDs). Among them, anorexia nervosa (AN) and bulimia nervosa (BN) are considered the most common ones both in the adult and young population [76]. Basically, both conditions are characterized by excessive attention to one’s weight, shape and attempts to manage weight: people who have AN are underweight in relation to their age and assigned gender at birth, engaging in actions to prevent weight growth. Patients with BN are characterized by multiple binge eating episodes and subsequent various weight compensatory behaviors, such as the use of diuretics or laxatives, high intensity physical exercise, fasting or self-induced vomiting [76]. As reviewed by Timko et al., recent literature shows how the proportion of males with EDs has increased, becoming much higher than previously considered; the female to male ratios reported in the last 10 years goes from 1:1 to 1:10 [13,77]. This has led to recent changes to the DSM-5 in order to make ED diagnostic criteria less gender specific (e.g., elimination of amenorrhea) [13]. Several studies agreed on the role of puberty in the development of EDs and attribute sex hormones as playing a significant role in this process: in this particular age, estrogen is described to worsen the risk in AFAB people [78,79]. Conversely, testosterone exposure during prenatal development seems to be a protective factor later in adolescence [80,81,82]. Moreover, some differences have been reported in the pattern of motives for restriction and food intake disorder: for instance, AMAB people tend to be more concerned with muscularity and shape than they are with body image or the desire to be thin [83,84]. Therefore, while they exercise more frequently to develop their muscles [85,86], AFAB people appear to more frequently express a wish to lose weight and look skinnier [87]. In addition to this, adolescent females with extreme EDs tend to also express suicidal thoughts and plans, while suicide attempts seem to be more frequent in their male peers [88].

Table 5 summarizes the available evidence on the relationship between EDs and gender.

## 9. Gender Diverse People and Neuropsychiatric Conditions

In the field of gender-based medicine, it should also be important to take care of gender nonconforming youths. Transgender (TG) is a term used to include people who cross define categories of gender based on the sex they were assigned at birth [89,90,91,92]; TG people may experience gender dysphoria (GD), which is defined as the discomfort that may come with the incongruence between one’s experienced gender and one’s assigned sex [91]. Recently, an American national survey has estimated that almost 0.73% of 13–17 year-old adolescents identify as TG [93].

Due to the onset of puberty and the possibility of experiencing significant psychological distress, adolescence is a particularly critical period for transgender youth. For example, a transgender male may experience breast development and menstruation, while a transgender female may experience voice changes, morning erections and hair growth [94]. The general assessment and specific medical care of youths with GD are driven by the World Professional Association for Transgender Health (WPATH), which has recently provided upgraded guidelines [2].

GnRH analogues (GnRHa), often known as puberty blockers, are frequently advised in the early stages of puberty to avoid or reduce dysphoria by preventing lasting changes to the body that misalign with recognized gender. A Tanner stage 2 of puberty is recommended in order to start pubertal suppression therapy: defined by the occurrence of breast budding in an assigned female at birth and by the attainment of a testicular volume of at least 4 mL in an assigned male at birth [2,95].

Gender-affirming hormone therapy (i.e., testosterone or estrogen) may follow GnRHa or not, with the target of producing physical transformations consistent with the identified gender. Studies reported that the correct management of TG adolescents within a multidisciplinary healthcare setting and timely treatment are associated with similar mental health outcomes observed in the general population [96]. A decrease in depressive symptoms, emotional problems and general functioning improvement were seen [97]. Moreover, Olson et al. highlighted similar mental health outcomes between socially transitioned prepubertal TG children and cisgender peers [98]

Recent studies have reported increased rates of gender diversity in the population of children and adolescents diagnosed with ASD compared to non-ASD peers (4–5.4% vs. 0.7%) [89,90,91,92,99]. Other studies conducted among children, adolescents and adults have identified a positive association between GD and ASD in a rate between 4.8 and 26% [100,101,102,103,104]. This may be due to the lower attitude to conform to societal norms frequently shown by individuals with ASD [105]. As reported by Butler et al., around 35% of young people referring to the Gender Identity Development Service of London present with moderate to severe ASD traits [106]. Nevertheless, as Warrier et al. have also highlighted in their review study, TG and gender diverse individuals do not necessary experience GD; therefore, it is likely a different rate of ASD incidence in GD youths compared to non-GD ones. Moreover, a high rate of ADHD, bipolar disorders, depression, learning disorders and schizophrenia is reported among TG and gender diverse individuals compared to cisgenders. In a recent study conducted among TG adolescents, 35% of youths reported suffering depression (11% in the severe-to-extreme range), 51% had presented suicidal thoughts and 30% of them had attempted suicide at least once [107]. In accordance with these results, it has been reported that TG teenagers who are subjected to bullying or physical or verbal abuse at school are more likely to suffer from anxiety problems and commit suicide [108]. These results are also supported by two other studies focused on depression, anxiety and substance abuse rates in TG people compared to cisgender people [109,110].

Regarding Eds, there is still lack of data, but as reported by Ferrucci et al., 5.6% of transgender people in a population of adolescents aged 12–15 years old received an eating disorder diagnosis [111]. This aligns with previous studies. Gender nonconforming youths are at higher risk of being exposed to social rejection, having unsupportive families and a lack of timely treatment or referral to specific care; it has been shown how this may contribute to the escalation of maladaptive behaviors, such as EDs [112]. It is notable that an eating disorder may also be the presenting symptom in adolescents with gender dysphoria demonstrating the value of addressing gender identity in youths with eating disorders [113].

Lately, it has been suggested that prenatal mechanisms involved in brain development, such as sex steroid hormone influence, may contribute to both neurodevelopmental conditions and gender role behavior, but further studies are still needed [114,115,116].

## 10. Conclusions

There is substantial evidence that gender disparities exist in neurologic and neuropsychiatric disorders among the pediatric population as already reported in adults [7]. Our literature analysis supports the fact that new guidelines should take this into account because there are still important gaps in the sector that need to be filled. Our findings suggest the need for specific studies focused on gender role among children and adolescents in order to better understand how this can relate to diagnosis, development and treatment of different neurologic and neuropsychiatric diseases. 

There is still a lack of data regarding the establishment of gender identity, but previous studies have highlighted how it seems to be a complex phenomenon in which epigenetic, biological and prenatal factors (including genes and hormones) could be involved [3,4,5]. Pediatric providers should consider the individual’s perception of being a male or female youth. As reported in a very recent review, sexual orientation is likely to be programmed into the brain during early development: a spike in testosterone masculinizes the unborn male brain during the intrauterine phase in the second half of pregnancy; in the absence of such a testosterone rush, a feminine brain will develop. Due to the fact that sexual differentiation of the genitals occurs far earlier in development than sexual differentiation of the brain, these two processes can be affected separately leading to gender dysphoria [4]. Moreover, no proof was observed that a person’s postnatal social environment has a significant influence on how they identify with one gender over another or with one sexual orientation [6]. All these considerations are urging us to direct further efforts in order to identify unique risks linked to gender disorders and gender dysphoria. A gender point of view should be taken into account when approaching a pediatric patient; this should include collecting information on gender identity in order to provide the best possible tailored heath care.

## Figures and Tables

**Table 1:** Available evidence on the relationship between febrile seizures (FS) and gender.

What is known
The prevalence of febrile seizure is significantly greater in AMAB people than AFAB (66% vs. 44%) [41,42]. The onset is earlier in AFAB people than in AMAB people (6–12 months vs. 36 months) [40].
What is still unknown
The neurobiological background of different gender-based prevalence of FS should be deeply investigated.
AFAB, assigned female at birth; AMAB, assigned male at birth.

**Table 2:** Available evidence on the relationship between anxiety/depression and gender.

What is known
Anxiety is one of the most common psychiatric disorders with adolescence onset [12,43,44]. In adolescence, an anxiety diagnosis is two times greater in AFAB people than in AMAB people [45]. In the adult age, the prevalence of major depressive episodes is two times higher in AFAB people than in AMAB people [45,54,55].
What is still unknown
The association between anxiety and brain structure abnormalities needs further investigations. It is not clear how the characteristics of depressive manifestations differ between AFAB and AMAB people.
AFAB, assigned female at birth; AMAB, assigned male at birth.

**Table 3:** Available evidence on the relationship between autism spectrum disorders (ASD), attention-deficit/hyperactivity disorder (ADHD) and gender.

What is known
The prevalence of ASD and ADHD is greater in AMAB people than in AFAB people (3–4 times and 2.4 times greater, respectively) [58,60,61,62,63,64,65,66]. Females show more serious symptoms related to ASD than males [65].
What is still unknown
ADHD sexual disparity factors are still uncertain. Studies show discordant data on neurobiological differences between AFAB and AMAB people diagnosed with ADHD.
AFAB, assigned female at birth; AMAB, assigned male at birth.

**Table 4:** Available evidence on the relationship between multiple sclerosis (MS) and gender.

What is known
The prevalence of MS is greater in AFAB people than in AMAB people [73,74,75].
What is still unknown
The role of sex hormones has been suggested for the onset of MS during adolescence, but further studies are needed. The relation between menarche and MS onset in young females must be deeply investigated.
AFAB, assigned female at birth; AMAB, assigned male at birth.

**Table 5:** Available evidence on the relationship between eating disorders (EDs) and gender.

What is known
Traditionally considered related to assigned female at birth, EDs are increasing among AMAB adolescents [13,77]. AMAB people tend to be more concerned about muscularity and AFAB people about losing weight. Suicide attempts are more frequent among AMAB people [83,84,85,86,87,88].
What is still unknown
The roles of puberty and of sex hormones seem to be critical to the onset of EDs but still need to be investigated. There is still a paucity of data regarding EDs among assigned males at birth.
AFAB, assigned female at birth; AMAB, assigned male at birth.

## Data Availability

Not applicable.
